# Stress among Emergency Health Care Workers on Nuclear or Radiation Disaster: A Preliminary State Survey

**DOI:** 10.3390/ijerph18168384

**Published:** 2021-08-08

**Authors:** Jean-Baptiste Bouillon-Minois, Vincent Roux, Bruno Pereira, Mara Flannery, Carole Pelissier, Céline Occelli, Jeannot Schmidt, Valentin Navel, Frédéric Dutheil

**Affiliations:** 1 Emergency Department, CHU Clermont-Ferrand, 63000 Clermont-Ferrand, France; jschmidt@chu-clermontferrand.fr; 2 CNRS, LaPSCo, Université Clermont Auvergne, Physiological and Psychosocial Stress, 63000 Clermont-Ferrand, France; vroux@chu-clermontferrand.fr (V.R.); fdutheil@chu-clermontferrand.fr (F.D.); 3 Clinical Research and Innovation Direction, CHU Clermont-Ferrand, 63000 Clermont-Ferrand, France; bpereira@chu-clermontferrand.fr; 4 Ronald O. Perelman Department of Emergency Medicine, NYU School of Medicine, New York University Langone Health, New York, NY 10016, USA; Mara.Flannery@nyulangone.org; 5 Service de Santé au Travail, CHU de Saint-Étienne, Saint-Étienne, France. Univ Lyon 1, Univ St Etienne, 42005 Saint-Étienne, France; carole.pelissier@chu-st-etienne.fr; 6 CHU Nice, Emergency Department, Université Côte d’Azur, 06000 Nice, France; celine.occelli@gmail.com; 7 Translational Approach to Epithelial Injury and Repair, Université Clermont Auvergne, CHU Clermont-Ferrand, CNRS, INSERM, GReD., 63000 Clermont-Ferrand, France; vnavel@chu-clermontferrand.fr; 8 Ophthalmology, CHU Clermont-Ferrand, 63000 Clermont-Ferrand, France; 9 Occupational and Environmental Medicine, CHU Clermont-Ferrand, WittyFit, 63000 Clermont-Ferrand, France

**Keywords:** disaster, emergency medicine, nuclear, radiation, prevention, public health, stress

## Abstract

Background: The nuclear or radiation disaster risk within the French Auvergne-Rhone-Alpes state is low (but not absent) due to its proximity to four Nuclear Power Generation Centers and two regional cancer control centers. This study aims to compare subjective stress ratings for emergency health care workers regarding nuclear and radiation disasters between two locations: at work versus at home. Materials and Methods: We distributed an anonymous online questionnaire via RedCap^®^ to all emergency health care workers who could be involved in patient care after a nuclear or radiation disaster. It comprised 18 questions divided into three parts—theoretical knowledge and practical assessment, stress assessment, and sociodemographic criteria. Results: We analyzed 107 responses. There was a significant 11-point increase in stress levels between work and home regarding nuclear or radiation disaster risks (*p* = 0.01). Less than 25% of emergency health care workers surveyed benefited from annual training. Conclusion: The stress levels of emergency health care workers regarding nuclear or radiation disaster were higher at work than at home and increased without annual training. It is important to increase knowledge about these protocols and to mandate yearly training for all workers potentially involved in these disasters.

## 1. Introduction

Since its discovery, the use of nuclear technology has increased both in the military and in society. France has the second-highest number of nuclear reactors after the United States, for a net electrical capacity of 61.4 GWe [1]. Since the inception of atomic science, preventative measures have been reinforced in all nuclear situations, mainly after each disaster [2]. The Institut de Radioprotection et de Sûreté Nucléaire (ISRN) has mandated policies regarding civil and military radioprotection in France since 2001 [3]. However, the risk is not absent, and a radiation accident such as Chernobyl [4] or Fukushima [5] is still possible. The Auvergne-Rhône-Alpes state has four nuclear power plants (Tricastin, Cruas, Bugey, and Saint-Alban) and two radiotherapy centers (Centre Jean-Perrin [6] and Centre Léon Berard [7]). Additionally, a putative terrorist attack with a radio-contaminated bomb is a conceivable scenario [8]. Furthermore, the mediatic impact and the fear generated in the population from such a scenario would be vast. In the last decade, France experienced numerous terrorist attacks, the most lethal of which were in November 2015 in Paris, [9] with 130 deaths and 413 injured, and in July 2016 in Nice, with 86 deaths and 458 injured [10]. As bio-terrorism is less frequent than plane crashes or gun violence and therefore has less of a mediatic impact, it is difficult to alert both the general population and health care workers to the risk of this putative disaster. Thus, proper characterization of potential radiation accidents is pertinent to circumventing the heavy burden placed on individuals exposed to and health care workers involved in these emergency responses, i.e., emergency health care workers (HCW). As those disasters are rare, with a very specific context in each situation, anticipated solutions are nearly impossible, and creativity and improvisation are crucial [11,12]. For more than two decades, studies have shown that stress is a factor that decision makers must contend with in most life-or-death situations. A better understanding of individual judgment and decision-making activities whilst under stress would yield a better understanding of how people make choices in emergencies [13]. Though planning, training, and technology can support improvisation, specific improvisations and “on the spot” ethical decisions cannot be anticipated or taken in advance [14].

In France, emergency medical dispatchers (EMDs) work with emergency physicians in a limited space in Service d’Aide Médicale Urgente (SAMU) [15]. Pre-hospital emergency HCWs are composed of firefighters (volunteers or professionals) with basic life support abilities, and mobile intensive care units (MICU) are composed of a certified driver, an emergency nurse, and an emergency physician working together to increase the quality of care [16]. Each site or hospital needs to have a hazardous material (HAZMAT) leader. In the event of a radio-contaminated accident, a particular unit—mobile radiologic intervention units (CMIR for Cellules Mobiles d’Interventions Radiologiques)—is present in each region. However, they are not dispatched at first but rather in a relay of first responders. Therefore, all emergency HCWs need to know both where to find personal protective equipment (PPE) and how to use it.

As all HCWs can potentially be involved in the care of patients in the event of a nuclear or radiological disaster, multiple dilemmas could be present. The main dilemma is whether to force participation (HCWs were impacted during previous disasters such as the Aum Shinrokyo Sarin attacks, where 10% of hospital HCWs involved in the care of exposed patients became patients themselves, mainly because of exposure to Sarin on other patients’ clothes) [14]. HCWs have an ethical dilemma between their obligations and their own safety [17]. Other dilemmas could include restricted access to the contaminated site by unauthorized people, forced restraint, gross criteria triage, assumed consent, forced decontamination, dignity, and assumed contamination [14]. For all of these reasons, nuclear or radiation disasters could be stressful in both everyday life and at work for first responders. Emergency HCW are a particularly at-risk population not only for both stress and burnout but also for suicide [18]. Indeed, their work is a complex interaction between stress due to life-threatening emergencies; overcrowding of the emergency department; lack of sleep; inadequate food intake before, during, and after shifts; and accumulated fatigue [19,20].

The main objective of our study was to assess subjective stress about nuclear or radiation disasters in emergency HCWs. The secondary outcomes were to assess the knowledge, theorical background, and training of firsts responders about nuclear and radiation disasters.

## 2. Materials and Methods

### 2.1. Study Location

This study was performed in the French state of Auvergne-Rhônes-Alpes. This state is composed of twelve departments. The main metropolitan center is Lyon, with 1.6 million inhabitants. There are four University Hospitals (Lyon, Grenoble, Saint-Etienne, and Clermont-Ferrand), for a total of 15 hospital groups.

### 2.2. Population

Any emergency HCW working in the state of Auvergne-Rhône-Alpes could enroll in our study as long as they would potentially be involved in patient care in the event of a nuclear or radiation disaster. We did not have any exclusion criteria except refusal to participate in the study.

### 2.3. Settings/Design

This was a multicenter, online, cross-sectional observational and descriptive study designed to assess stress among emergency HCWs in the case of a nuclear of radiological disaster.

The primary objective was the assessment of stress regarding nuclear or radiological disasters at work and at home among emergency HCWs. The secondary objectives were to assess the knowledge, theorical background, and training of first responders about nuclear and radiation disasters. The investigators initiated and distributed an anonymous online questionnaire. Completion of the survey took approximately five minutes per participant.

### 2.4. Questionnaire

We did not find a survey representative of the French population of emergency HCWs, so we created our own 18-question survey. The first section was two visual analogic scales (VASs) assessing stress about nuclear or radiation disasters (main outcome) at home and at work. VASs assess stress on non-calibrated horizontal lines 10 cm in length, ranging from no stress (0) to the maximum level of stress (100). VASs are proven to be at least as discriminating as a questionnaire in highlighting differences in stress levels, and the correlations observed with related constructs support its construct validity [21,22]. The second section was about knowledge, training, and ability to form theories. We also used visual analog scales to quantify the ability to diagnose hazardous material exposure of a patient. The third part comprised sociodemographic questions (sex, age, work, and experience).

### 2.5. Data Collection

The survey was distributed, captured, and stored in RedCap^®^ (software with an institutional account in the University Hospital of Clermont-Ferrand, Clermont-Ferrand, France). A QR code was created and sent via e-mail to every first responder in the Auvergne-Rhone-Alpes state. All data were anonymous, with no possibility of linking a response to a particular participant after survey completion.

### 2.6. Statistical Analysis

This was a preliminary study planned to assess stress levels between two situations (home vs. work) in emergency HCWs. We hypothesized three points (with 10 points of standard deviation) of VAS difference between stress at home and stress at work inducing a minimal number of subjects to 90 people [23]. We also hypothesized that the impact of sociodemographic and background would increase stress by 25 points (considered high impact) between stress at home and stress at work. A statistical analysis was performed according to the Strengthening the Reporting of Observational Studies in Epidemiology (STROBE) checklist [24]. The results were descriptive in nature and those for all closed questions were provided in absolute numbers or percentages. The responses to each questionnaire were recorded into RedCap^®^, and extraction was performed to a Microsoft Excel database and analyzed using Stata^®^ software version 16 (StataCorp, College Station, Texas, USA). The categorical variables were expressed as frequencies and associated percentages. Continuous data were expressed as mean ± standard deviation or median [interquartile range]. To measure the increase in stress between stress at home and stress at work, paired t-tests were performed. Furthermore, to examine the impact of different parameters (training, theorical background, and sociodemographic), the ANOVA and Kruskal–Wallis tests were used. All statistical tests were performed for a two-sided type I error of 5%.

### 2.7. Ethical Considerations

This study is a part of the SEEK protocol, a novel protocol assessing stress among emergency health care workers [25]. A French Ethics committee (Comité de Protection de Personnes Sud-Est I, CHU Saint-Etienne) approved this study protocol on 3 November 2014 (reference DC-2014-2151), and the protocol was registered in ClinicalTrials (NCT02401607).

## 3. Results

### 3.1. Characteristics of the Population

Between May 1st and May 31st, 2021, a total of 107 questionnaires were recorded and analyzed. The respondents were equally gender-distributed (49.5% women). The mean age of our group was 39.5 ± 4.5 years old, with most respondents (69%) between 26 and 45 years of age. The occupations of respondents were mostly nurses (42.1%), followed by physicians (29.9%), professional firefighters (25.2%), volunteer firefighters (6.5%), and ambulance drivers in the MICU (3.7%). Eleven responders (10.3%) were also leaders for hazardous material disasters in their own center. Eight physicians (7.5%) worked at their SAMU center (Table 1).

### 3.2. Main Objective

Among the 107 respondents, mean stress regarding a nuclear or radiation disaster at work (31.3, 95CI 25.9 to 36.6) was significantly higher than mean stress with regard to nuclear or radiation disaster at home (19.3, 95CI 14.6 to 23.9) (*p* = 0.01). There were also more individuals with stress levels higher than 50/100 at work than at home (23.4% vs. 13.1%, *p* < 0.001).

### 3.3. Secondary Objectives

#### 3.3.1. Diagnosis

Among the 107 responders, mean VAS was 36.8 +/− 26.5 (between 10 and 94) for their ability to diagnose exposure to a hazardous material in a patient. We found a positive impact of being a HAZMAT referent for diagnosis (*p* < 0.001). Indeed, the VASs were 67 +/− 20.3 among eleven HAZMAT referents and 33.3 +/− 24.9 among non-HAZMAT referents. In all responders, 81 (75.7%) were able to cite hematologic consequences, 84 (78.5%) cited dermic consequences, 75 (70.1%) cited digestive consequences, and 48 (44.9%) cited neurological consequences.

#### 3.3.2. Appropriate Contact

In the event of nuclear or radiation exposure in a patient, 90 responders (84.1%) would ask the emergency physician in SAMU for guidance, 10 (9.3%) would ask the HAZMAT referent in their center, six (5.6%) would ask a radiologist, and one would ask the army (0.9%).

#### 3.3.3. Theoretical Knowledge/Training

**Figure 1:**
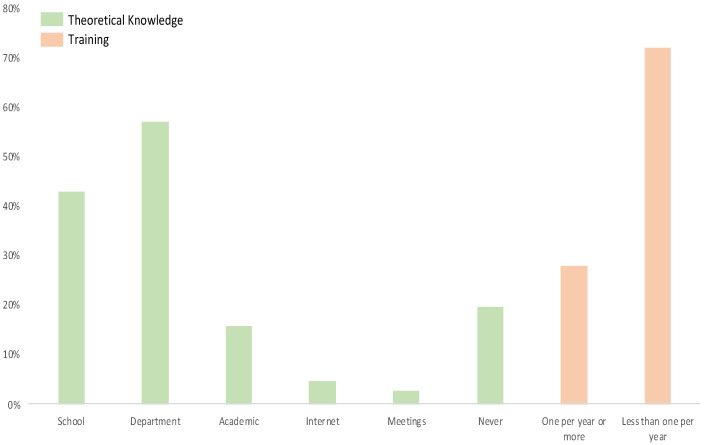
Theoretical knowledge/background.

Among 107 responders, 61 (57%) reported no theoretical lessons during school (medical school, nursing school, driving school, or firefighter training programs), 61 (57%) reported that they were taught in their hospital or fire department, 17 (15.9%) had official training after school, five (4.7%) were trained during a conference, and 21 (19.6%) declared that they had never had any theoretical lessons on HAZMAT. Thirty-seven (34.6%) never had any training on a HAZMAT disaster, 38 (35.5%) had less than one per year, ten (9.4%) had one per year, and only 22 (20.6%) had more than one per year (Figure 1). Lastly, 33 (30.8%) declared that they did not know what type of personal protective equipment (PPE) was mandatory in the event of a HAZMAT disaster. Eleven respondents (14.9%) did not know where the PPE at their workplace was kept.

#### 3.3.4. Factors Influencing Perceived Stress

**Figure 2:**
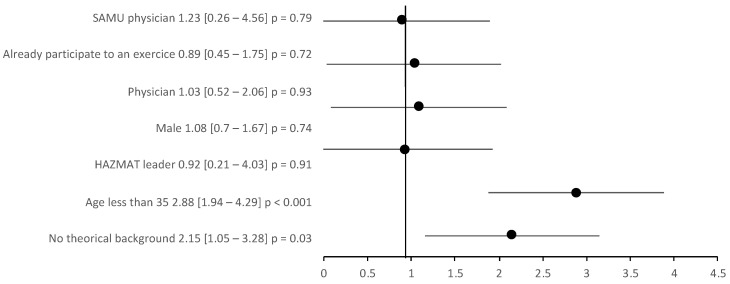
Risk ratio increase in the stress visual analog scale by >25 points.

We found a significant impact on VAS stress (up to 25 points) in the case of the absence of theoretical background (risk ratio = 2.15, CI95% 1.05–3.28, *p* = 0.01), and an age of 35 years old or younger (RR 2.88, 95% CI 1.94–4.29, *p* < 0.001). However, there was no protective effect or risk factor associated with being a HAZMAT leader (RR = 0.92, 95% CI 0.21–4.03, *p* = 0.91); a physician (RR = 1.03, 95% CI 0.52–2.06, *p* = 0.93); male (RR = 1.08, 95% CI 0.7–1.67, *p* = 0.74); and, more interestingly, a physician working in the SAMU (RR = 1.23, 95% CI 0.45–1.73, *p* = 0.94) or in responders that already had training (RR = 0.89, 95% CI 0.45–1.75, *p* = 0.45) ( Figure 2).

## 4. Discussion

We found a positive increase in subjective stress assessed by VAS concerning nuclear and radiation disasters between home and the workplace in emergency health care providers.

Stress has historically been assessed with a complete survey such as Cohen’s Perceived Stress Scale [19]. However, because of survey length, many were not completed. Although it was first developed to assess pain in medicine, the use of a VAS is very attractive because of its simplicity and reproducibility [21,22]. VASs were studied and validated to determine the stress on the general population. Furthermore, VASs are less vulnerable to confounding factors than a Likert scale in stressed patients [26], and it is now a standard tool used by occupational physicians to assess stress among workers [21].

We also demonstrated that stress levels regarding nuclear and radiological disasters at home are very low (19.3, 95CI 14.6 to 23.9) and are significantly lower than when at work. Even if it was expected, these findings are novel in that they demonstrate that emergency HCWs were able to relax when they were not on call. Studies have shown that, when stress at work is high, it tends to be worse outside of working hours but significantly lower after working [27]. The impact of rest periods in shift workers such as HCWs were studied across more than two decades and showed a positive impact of 12-h shifts on wellbeing, mainly because of the increase in rest periods [28]. Despite the relation between stress at work and at home being poorly studied, our study showed that emergency HCWs are able to rest when they are not on call. This has potential positive effects on burnout, wellbeing, anxiety, stress, and cardiovascular disease [29].

Emergency HCWs are on the front line for all HAZMAT disasters. Fortunately, HAZMAT disasters are rare, and nuclear and radiation disasters are even more infrequent. However, the only way to lessen the impact of a nuclear or radiation disaster is to train all emergency HCWs involved in this type of incident. Unfortunately, our survey showed that not all receive a robust education and annual training. Although it was not our main objective, this point is very relevant. All emergency HCWs must have significant theoretical knowledge and at least one specific training regarding local risks. Indeed, training sessions have shown a positive effect on many situations [30], even including disaster preparedness [31]. Our results are in line with that in the literature. Indeed, although physicians indicated the occurrence of a terrorist or bioterrorist attack, and chemical, railway, and/or air disasters as being possible in Poland, they perceived their disaster preparedness as being insufficient [32]. Furthermore, although one study has shown that there are no statistically significant differences in the duration of orotracheal intubation with or without HAZMAT PPE, this study was only performed with trained physicians. However, our study showed that 15% of responders do not even know where the PPE equipment is located in their center [33]. Some indicators have been proposed for HAZMAT training and knowledge, such as training for hazmat team members, mandatory equipment for teams, response plans, medical surveillance programs, hazmat team structures, incident command systems, hazmat team qualification of different levels, and certification and maintaining that certificate [34].

These findings on nuclear or radiological disasters are in accordance with that in the literature on biological disasters. In Pakistan, one of the most heavily armed nuclear zones in the world, a study showed an inadequate level of preparedness for a nuclear, biological, or chemical incident and proposed seminars for undergraduate medical and nursing students [35]. Furthermore, three years after the Severe Acute Respiratory Syndrome (SARS) outbreak, Canadian nurses were asked to assess their sense of preparedness for infectious disease outbreaks and for chemical, biological, radiological, and nuclear disasters associated with terrorist attacks. The results were that 40% of nurses were unsure if their hospital had an emergency plan for a large-scale outbreak. They also reported inadequate access to resources to support disaster response capacity and expressed a low degree of confidence in the preparedness of Canadian health care institutions for future outbreaks [36]. Each hospital has a HAZMAT referent who is an emergency physician. Emergency HCWs are well known for having great sense of responsibility for their work, a characteristic recently proven during the COVID-19 pandemic [37]. Even in countries where a lockdown was efficient [38,39], emergency HCWs reduced the incidence levels of COVID-19 patients being transferred to intensive care units. Emergency HCWs are also a population with an increased incidence of burnout and job strain [20,25]. It is very important to consider HCWs both as helpers and victims after a disaster [40]. One recent study proposed guidelines to protect HCWs during the COVID-19 pandemic [41].

In France, emergency physicians are available at the call center reception—i.e., the SAMU—24 hours a day. They are aware of all calls from all patients and determine the best destination for all patients according to their level of disease, to the technical equipment of local hospitals, and to overcrowding in emergency departments. They are also a referent on call in case of any doubt regarding pre-hospital management, and our study confirmed that characteristic. Indeed, if only 30% of responders were physicians, more than 80% considered the emergency physician on call in the SAMU center as the referent for all questions regarding nuclear or radiation disasters. As the referent, the SAMU physician needs to be firm in their decision. They must decide very rapidly among competing courses of action, none of which is without negative consequences [14]. This burden on the SAMU’s shoulders is a risk factor for post-traumatic stress disease. Very interestingly, being a SAMU referent was not a criterion for being less stressed than a non-SAMU physician. We also proved that emergency HCWs do not have sufficient knowledge on the theoretical diagnosis of nuclear and radiational exposure. These data are in line with that in the literature [42].

Our study has some limitations. First, it is a new survey that could not be compared with any other found in the literature. Second, the short period of recruitment allowed us to only gather responses from a negligible portion of the population. However, it was sufficient to obtain a significant difference between stress at work and stress at home regarding nuclear and radiational disasters. Third, our survey was performed in a French state with a very strict and robust policy regarding all nuclear and radiation instruments. Even if the risk is not absent, it is quite improbable that such an event would occur. This could explain the possible low level of stress found in our responders. However, we asked all respondents to answer with as much relevance as possible to such an event occurring. Furthermore, this study is a preliminary one with a small sample size. We plan to perform the same survey in different areas with different levels of nuclear and radiological risks.

## 5. Conclusions

Nuclear and radiation disaster risk is very low in the French state of Auvergne-Rhone Alpes mainly because of the nuclear and radiation safety protocols. However, the risk is not absent. All emergency HCWs can potentially be involved in the care of patients exposed to that type of disaster. However, we found that more than 57% have never received any theorical instruction and that only 30% are involved in annual training. We assessed subjective stress using a visual analog scale both at home and at work. The results showed a significantly higher level of stress at work versus at home regarding nuclear or radiation disaster in emergency HCWs. This increase is even more significant in those 35 years old and under who have no theorical background or annual training. It is important to increase knowledge about these protocols and to organize yearly training for all workers potentially involved in this type of disaster. It would also be interesting to perform the same study for states with a higher number of emergency HCWs and in areas with a higher risk of disaster.

## Figures and Tables

**Table 1:** Sociodemographics. SD = standard deviation, HAZMAT = hazardous materials, SAMU = Service d’Aide Médicale Urgente.

Characteristics	Mean	SD	*n*	%
**Age (years)**	39.5	4.5		-
<25	-	-	2	1.9
26–35	-	-	38	35.5
36–45	-	-	35	32.7
46–55	-	-	24	22.4
>55	-	-	8	7.5
**Sex**				
Female	-	-	53	49.5
Male	-	-	54	50.5
**Work**				
Ambulance driver	-	-	4	3.7
Nurse	-	-	45	42.1
Physician	-	-	32	29.9
Volunteer firefighter	-	-	7	6.5
Professional firefighter	-	-	27	25.2
HAZMAT leader	-	-	11	10.3
SAMU Physician	-	-	8	7.5

## Data Availability

The data presented in this study are available on request from the corresponding author.
